# Oxidative Stress and Neurodegeneration in Animal Models of Seizures and Epilepsy

**DOI:** 10.3390/antiox12051049

**Published:** 2023-05-05

**Authors:** Krzysztof Łukawski, Stanisław J. Czuczwar

**Affiliations:** 1 Department of Physiopathology, Institute of Rural Health, Jaczewskiego 2, 20-090 Lublin, Poland; lukaw@mp.pl; 2 Department of Pathophysiology, Medical University of Lublin, Jaczewskiego 8b, 20-090 Lublin, Poland

**Keywords:** free radicals, oxidative stress, epileptogenesis, epilepsy, seizures, antioxidants

## Abstract

Free radicals are generated in the brain, as well as in other organs, and their production is proportional to the brain activity. Due to its low antioxidant capacity, the brain is particularly sensitive to free radical damage, which may affect lipids, nucleic acids, and proteins. The available evidence clearly points to a role for oxidative stress in neuronal death and pathophysiology of epileptogenesis and epilepsy. The present review is devoted to the generation of free radicals in some animal models of seizures and epilepsy and the consequences of oxidative stress, such as DNA or mitochondrial damage leading to neurodegeneration. Additionally, antioxidant properties of antiepileptic (antiseizure) drugs and a possible use of antioxidant drugs or compounds in patients with epilepsy are reviewed. In numerous seizure models, the brain concentration of free radicals was significantly elevated. Some antiepileptic drugs may inhibit these effects; for example, valproate reduced the increase in brain malondialdehyde (a marker of lipid peroxidation) concentration induced by electroconvulsions. In the pentylenetetrazol model, valproate prevented the reduced glutathione concentration and an increase in brain lipid peroxidation products. The scarce clinical data indicate that some antioxidants (melatonin, selenium, vitamin E) may be recommended as adjuvants for patients with drug-resistant epilepsy.

## 1. Introduction

When the steady-state balance of pro-oxidants to antioxidants alters in favor of the former, the phenomenon known as oxidative stress occurs, raising the possibility of organic damage [1]. ROS (reactive oxygen species), ROI (reactive oxygen intermediates), and RNS (reactive nitrogen species) characterize an expanding family of endogenous, highly reactive, oxygen- (and also nitrogen-) carrying compounds [2]. ROS may occur as free radicals (atoms or groups of atoms that serve as electron acceptors and have an unpaired electron) and non-radical reactive species [3,4]. ROS include both oxygen radicals such as superoxide, O_2_^•−^, hydroxyl, HO^•^, or hydroperoxyl, HO_2_^•^, and certain non-radicals that are oxidizing agents and/or are easily converted into radicals (e.g., hydrogen peroxide, H_2_O_2,_ hypochlorous acid, HOCl, hypobromous acid, HOBr, peroxynitrite, ONOO^−^, or Ozone, O_3_) [3]. The most prevalent source of ROS is the subsequent one-electron reduction of oxygen. Such a reduction of oxygen, for example, creates superoxide radical anions, O_2_^•−^, while one-electron reduction of H_2_O_2_ yields hydroxyl radicals, HO^•^ [5]. The hydroxyl radical is the most damaging free radical to cells [1]. RNS (reactive nitrogen species) is a similar collective term to ROS and includes nitric oxide, NO^•^, nitrogen dioxide, NO_2_^•^ and nitrate, NO_3_^•^, as well as non-radicals such as nitrous acid, HNO_2_, and dinitrogen tetroxide, N_2_O_4_ [3]. Peroxynitrite (ONOO^−^) is also classifiable as an RNS [3].

The effects of oxidative stress on the organism are dependent on the type of oxidant, the location and intensity of its production, the composition and activities of various antioxidants, and the ability of repair mechanisms [6]. Mitochondria are the primary producers of ROS in cells, and the enzymes in the electron transport chain, in particular, play a significant role in this process [5]. Mitochondria are highly vulnerable to oxidative damage since they are the primary source of ROS generation [1]. Other sources of ROS production involve peroxisomal oxidases, cytochrome P-450 enzymes, NADPH oxidase, or xanthine oxidase [6]. Even under physiological conditions, the production of ROS and RNS is inevitable [7], and the brain is a good example of this activity [4]. Furthermore, the brain generation of ROS is highly comparable to other organs and greatly increases during brain activity [4]. Because of its low overall antioxidant capacity, the brain is especially vulnerable to free radical damage. ROS generated in brain tissue can cause direct damage to macromolecules such as lipids, nucleic acids, and proteins [6]. NADPH (nicotinamide adenine dinucleotide phosphate) oxidase (NOX), mitochondria, xanthine oxidase, and lipoxygenase are the major ROS producers in the brain, and, in turn, the two main endogenous antioxidants in lipids, reduced glutathione (GSH) and α-tocopherol (vitamin E), perform significant protective functions together with antioxidant enzymes [4]. NADPH is required for the reduction of oxidized glutathione (GSSG) to GSH, a reaction catalyzed by glutathione reductase, and GSH is necessary to protect the sulfhydryl groups of proteins and to prevent peroxidation of lipids in the membrane [8]. In other words, GSH protects against lipid peroxidation (LPO) in a tissue, which is an indicator of permanent biological damage to the cell membrane phospholipid, which, in turn, leads to inhibition of most of the sulfhydryl and some nonsulfhydryl enzymes [9]. A rise in malondialdehyde (MDA) levels is a marker of LPO [10]. Vitamin E interrupts free radical chain reactions by transferring phenolic hydrogens to peroxyl free radicals of peroxidized polyunsaturated fatty acids (PUFAs) found in cellular and subcellular membrane phospholipids, acting as the first line of defense against peroxidation of PUFAs [8]. Apart from a non-enzymatic antioxidant defense system including reduced GSH and vitamins E and C, physiological levels of ROS can be scavenged by antioxidant enzymes (e.g., superoxide dismutase—SOD, catalase—CAT, glutathione peroxidase—GPx, glutathione reductase—GR, and peroxiredoxins—Prxs) [11]. Excessive ROS levels cause oxidative stress as a result of increased ROS generation and impaired antioxidant defense capabilities, and additionally react with nitric oxide to generate RNS including very reactive ONOO^−^ [11]. Because excessive levels of nitric oxide and its oxidative derivatives, such as ONOO-, can be toxic and play an important role in neurodegenerative diseases, nitrite content is also regarded as a biomarker of oxidative stress [9]. There is a steady-state equilibrium between the generation of nitric oxide and its metabolites (nitrite and nitrate) and their elimination by antioxidant mechanisms under normal conditions [9]. Oxidative stress, induced by the imbalance between excessive ROS formation and limited antioxidant defense, has been linked to a variety of pathologies, e.g., age-related disorders (cardiovascular, cancer, inflammatory) and neurodegenerative diseases such as Parkinson’s and Alzheimer’s diseases [2].

## 2. Epileptic Seizures and Epileptogenesis

**Figure 1:**
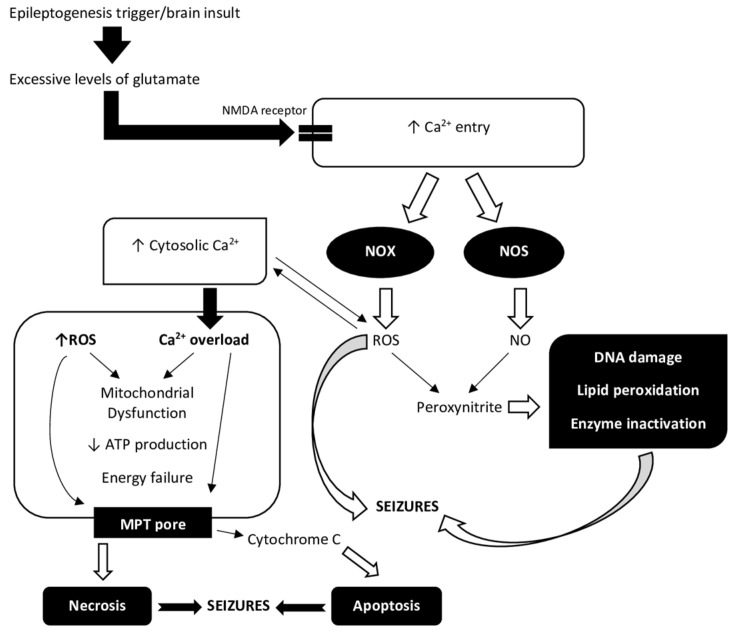
A relationship between Ca^2+^ ions and reactive oxygen species (ROS) in the process of epileptogenesis. Increased extracellular glutamate concentrations after brain insult have been related to a variety of processes, including excessive glutamate release and impaired cellular uptake. Increased levels of glutamate in the extracellular compartment are likely to be toxic to neurons. Excitotoxicity causes an excessive activation of glutamate receptors, namely NMDA (N-methyl-D-aspartate) and AMPA (α-amino-3-hydroxy-5-methyl-4-isoxazolepropionic acid) receptors, and a corresponding massive rise in free cytosolic and mitochondrial Ca^2+^ concentration [22]. Oxidative stress during and after seizures plays a role in both immediate and long-term excitotoxic neuronal death. An epileptogenesis trigger, such as status epilepticus (SE), causes Ca^2+^ entry via NMDA receptors. NADPH oxidase (NOX) and nitric oxide synthase (NOS) are two of the enzymes that are activated by Ca^2+^ entry and NMDA receptor activation. Peroxynitrite is formed by ROS and nitric oxide (NO) and is toxic to DNA, proteins, and lipids [4]. ROS regulate the activity of redox-sensitive enzymes and ion channels, including Ca^2+^ channels, within the cell. There is a delicate balance between the beneficial and harmful effects of Ca^2+^ and ROS on mitochondria [23]. Elevated Ca^2+^ concentration in the cytosol leading to over-stimulation of Ca^2+^ signaling pathways results in mitochondrial dysfunction. Mitochondria take up Ca^2+^ ions from the cytosol, which overload the mitochondria and, together with ROS, reduce ATP production and cause energy depletion. Complexes I, II, III, IV, and V form the mitochondrial respiratory chain. ROS can react with thiol groups (-SH), opening the mitochondrial permeability transition (MPT) pore as a result [22]. This further interferes with mitochondrial function and also allows cytochrome c into the cytosol, where it can trigger apoptotic pathways [4]. Ca^2+^ uptake, mitochondrial Ca^2+^ overload, and ROS generation can also cause necrosis due to mitochondrial damage [23]. Both processes are involved in epileptogenesis.

A growing body of experimental data suggests that oxidative stress may play a role in the pathophysiology of seizures, epilepsy, and epileptogenesis. Excessive production of ROS during seizures causes LPO, DNA damage, enzyme inhibition, and mitochondrial damage, ultimately leading to neuronal death [4]. Epileptic seizures, “a transient occurrence of signs and/or symptoms due to abnormal excessive or synchronous neuronal activity in the brain” [12], are generally classified as either focal or generalized epileptic spasms [13]. Focal seizures (formerly known as partial seizures) originate within networks that are restricted to one cerebral hemisphere and can be discretely localized or more broadly spread [13,14]. Generalized epileptic seizures can affect cortical and subcortical regions, although not always the entire cortex. They originate within bilaterally dispersed networks that become quickly involved [13]. Epilepsy is the most common serious chronic neurological disease, and affects 65 million people worldwide [14]. It is often assumed that around 70% of people with epilepsy can be sufficiently controlled with available antiepileptic drugs (AEDs), also known as antiseizure drugs, while the other 20–30% are drug-resistant, which is likely to be underestimated [15]. Epileptogenesis is defined as the development and extension of tissue capable of generating spontaneous seizures, resulting in the development of an epileptic condition and/or progression of epilepsy after it is established [16]. Traumatic brain injury (TBI), infections, stroke, tumors, craniotomy, neurodegenerative disorders, cerebral palsy, intrapartum hypoxia, and prolonged acute symptomatic seizures, including complex febrile seizures and status epilepticus (SE), have all been shown to induce epileptogenesis [17,18]. The exact mechanism by which an initial brain insult causes epilepsy is still unknown. However, glutamate excitotoxicity, neuroinflammation, and oxidative stress seem to represent a pathogenic “triad” that characterizes the neurobiology of various brain disorders, including epilepsy, leading to seizure-induced cell death, increased susceptibility to neuronal synchronization, and network alterations [19]. Increased ROS production and oxidative stress seem to be common characteristics of surgical specimens from chronic epilepsy patients as well as experimental epileptogenic insults [20]. Like inflammation, elevated ROS is thought to be both a cause and consequence of seizures [21]. It appears that inflammation and ROS production are closely related. ROS production is modulated by inflammatory pathways, while inflammatory processes are modulated by ROS [20]. It has been proposed that oxidative stress is a prominent result of glutamate excitotoxicity, which plays a critical role in epileptic brain injury [22]. Following epileptogenic brain insult, excessive ROS generation increases the concentration of Ca^2+^ ions via mechanisms outlined in Figure 1. Increased concentration of intracellular Ca^2+^ leads to overloading of mitochondria with Ca^2+^ ions. Although there is a balance between beneficial and detrimental effects of Ca^2+^ and ROS on mitochondria [23], Ca^2+^ overload may be involved in seizure-evoked neuronal death, as well as in necrosis and apoptosis [22].

This review shows the evidence for the generation and consequences of oxidative stress in numerous animal models of epilepsy, including the damage to proteins, lipids, and antioxidant defense. Because neuronal cells are thought to be particularly vulnerable to oxidative damage, and neuronal cell death is a contributing factor in the development of epileptogenesis, we give attention to oxidative stress in connection to cell death.

## 3. Short Description of Animal Models of Seizures, Epilepsy and Epileptogenesis

For decades, animal models of acute seizures evoked electrically or chemically in naive animals, such as the maximal electroshock seizure test (MES) and subcutaneous pentylenetetrazol seizure test (scPTZ), have been used to investigate the anticonvulsant potential of novel drugs. In conjunction with the classical kindling model, a model with chronic seizures brought on by repeated electrical stimulation of the amygdala or hippocampus in rats, these models predicted the anticonvulsant efficacy of all clinically used AEDs in patients with epilepsy [24]. In the identification step of a potential anticonvulsant compound, a 6 Hz psychomotor seizure model is also employed [25]. Once the effectiveness of an investigational AED is demonstrated using one of the acute seizure models (MES, scPTZ, or 6 Hz seizure test), subsequent tests are performed to assess the active compound’s anticonvulsant potency [26]. These include tests such as the classical amygdala- or hippocampus-kindled rat, audiogenic seizures, and different chemical models of seizures [26,27]. Multiple chemical models of seizures and epilepsy (e.g., bicuculline, picrotoxin, strychnine, kainate, pilocarpine, and other cholinergic agents) have been introduced [28]. Some of them have a predictive value concerning the clinical profile of efficacy of tested anticonvulsants, and some may contribute to a better understanding of possible mechanisms of epileptogenesis [28]. A chemical kindling method using PTZ has also been applied for the evaluation of novel anticonvulsants in chronic seizures [29].

Several existing animal models of epilepsy have been used in studies on epileptogenesis. Among them are the above-mentioned kindling models, including the PTZ kindling model [29]. Particularly, the amygdala- or hippocampus-kindled rat is a useful tool in this respect [30]. Other models widely used for studying epileptogenesis include post-status epilepticus (SE) rodent models of temporal lobe epilepsy (TLE), such as pilocarpine and kainate [30,31]. In these models, the chemoconvulsant-induced SE is followed by spontaneous recurrent convulsions after a latent period of days to weeks, and drugs with possible antiepileptogenic activity are administered during the latent period [31]. TBI (traumatic brain injury) and stroke models, models of febrile seizures, and genetic models of epilepsy are other experimental models of epileptogenesis [31,32].

In recent years, many animal tests have been discussed as models of drug-resistant epilepsy, which can be categorized into the following groups: models of seizures with a poor response to different AEDs (e.g., 6 Hz psychomotor seizure model, lamotrigine-resistant kindled rat), models that choose certain subgroups of drug-resistant animals (e.g., phenytoin-resistant kindled rat), and models in which resistance evolves over time (e.g., PTZ or 6 Hz seizure test in epileptic rodents) [33].

## 4. Seizures and Oxidative Stress: Evidence from Animal Models of Seizures, Epilepsy and Epileptogenesis

### 4.1. Maximal Electroshock Seizure Models

Tonic–clonic seizures are generated by bilateral transauricular or corneal electrical stimulation in the MES test [27]. The MES test is thought to be a reasonable model of generalized tonic–clonic seizures in humans [27]. Electroconvulsive shock-induced generalized seizures in animals have been also used as a common model of electroconvulsive therapy in humans [34]. An increase in LPO was reported for electroshock-induced seizures as determined in the whole brains of mice or rats [35,36,37,38], or in some brain structures, especially in the cortex [34,39,40]. A long-lasting increase in LPO has been also demonstrated in the hippocampus and striatum after multiple electroshock [41]. On the other hand, following electroconvulsions, a reduction in LPO was observed in the hippocampus, striatum, and cerebellum [39,42], indicating that electroshock-induced seizures cause brain structure-specific alterations in the levels of LPO [39]. Some studies showed that, after electroconvulsions, a change in the level of LPO could occur in the brain, and this may be connected with the generation and elimination of free radicals. After MES-induced seizures, LPO decreased immediately, however, its statistically significant rise was visible three hours later in the brain tissue [43]. It was hypothesized that LPO is at some stage prevented by a high level of GPx activity, which detoxificates H_2_O_2_ and prevents oxidative damage. The later increase in LPO in the brain is dependent on the decrease in GPx activity [43]. In turn, a decrease in LPO in the rat hippocampus immediately after and up to 30 days after a single or multiple electroconvulsive shocks has been demonstrated, indicating no oxidative damage in this structure [42]. In the same study, an increase in catalase (CAT) and superoxide dismutase (SOD) activities at different time points after single and multiple electroconvulsive shocks was observed in the hippocampal tissue [42]. The increases in SOD and GPx activities in the hippocampus and cerebellum of rats were also observed in other studies, suggesting structure-related changes in the activities of antioxidant enzymes following electroconvulsions [34]. On the other hand, numerous studies have shown that electroshock-induced seizures rather decrease antioxidant enzymes, SOD, GPx, glutathione reductase (GR), or CAT, as measured in the whole brain [36,38] or in various individual brain regions, i.e., the frontal cortex, hippocampus, cerebellum, pons-medulla, and striatum [41,44]. It has been demonstrated that some brain structures exhibited a delayed rise in oxidative damage following electroshock and a reduction in antioxidant enzyme activities [41,44]. Additionally, it has been suggested that elevations in some antioxidant enzymes (SOD and GPx) in specific brain regions (e.g., hippocampus) could be an immediate compensatory adaptation of the free-radical scavenging enzymes for the electroshock-induced increase in ROS production [34]. SOD metabolizes the excess of O_2_^•−^ and produces H_2_O_2_ which is then transformed to H_2_O via GPx action. The balance between SOD on one hand and GPx and CAT on the other is more significant for free radical conversion than the activity of a single antioxidant enzyme [34]. Furthermore, based on current research, it can be suggested that oxidative damage following electroconvulsions, as measured by LPO and antioxidant enzyme activities, may be connected to the type of electroshock (i.e., acute vs. chronic) and its intensity.

### 4.2. Pentylenetetrazol-Induced Seizures

PTZ-induced clonic convulsions are induced by subcutaneous or intraperitoneal injection of PTZ, a GABA_A_-receptor antagonist, at convulsant doses [27]. The PTZ test is thought to be a model of generalized myoclonic or absence seizures [27]. As with electroconvulsions, an increase in LPO has been observed in the brains of animals after acute pentylenetetrazole (PTZ) injection when determined in whole brains [36,37,38] or in different brain structures measured separately, i.e., in the hippocampus [45,46,47], cortex [47,48,49], and striatum [47]. Numerous studies revealed that the activities of antioxidant enzymes, SOD, GPx, glutathione reductase (GR), and CAT, were reduced in the rat or mouse brains due to seizures induced by PTZ [36,38,46,50]. Acute PTZ administration causes region-specific biochemical alterations, and major changes in enzyme activities have been observed in the cerebral cortex and hippocampus [50]. Researchers have tried to explain the unchanged SOD activity in the cortex of PTZ-treated animals as being caused by simultaneous upregulation of SOD and enzyme degradation caused by oxidative stress [50]. Patsoukis et al. [51,52] examined the thiol redox state (TRS) components (glutathione (GSH), glutathione disulfide (GSSG), cysteine (CSH), protein (P) thiols (PSH), and protein and non-protein (NP) mixed/symmetric disulfides (PSSR, NPSSR, NPSSC, PSSP)) in mouse cerebral cortex and hippocampus following PTZ-induced seizures. A significant decrease in GSH, GSSG, CSH, NPSSC, PSSR, and PSSC, as well as an increase in protein carbonyl and a high increase in PSSP levels, were found in the cerebral cortex, indicating increased oxidative stress [51]. In the hippocampus, PTZ convulsions resulted in a decrease in PSH, CSH, and NPSSC, as well as an increase in PSSP, NPSSR, LPO, and protein oxidation levels, indicating increased oxidative damage in this structure [52]. Another study reported a reduction in the levels of GSH and antioxidant enzymes in the mouse hippocampus, with concurrent elevation in the hippocampal level of NOx (nitrate/nitrite), an indicator of nitric oxide production and 3-NT (3-nitro-tyrosine), a marker for nitration of protein, elevated immune expression of NF-κB activity, and lowered NF-E2-related factor 2 (Nrf2) activity [46]. It should be added that NF-κB is a trigger for the expression of various inflammatory mediators and ROS generation, while Nrf2 can regulate various antioxidant enzymes at the transcriptional level [46]. Dramatic fivefold elevation of nitric oxide production following PTZ-induced seizures was found in the cerebral cortex in rats [48]. However, the role of nitric oxide generation in PTZ convulsions is still a matter of controversy, with reports showing either pro- or anticonvulsant effects of nitric oxide in this type of seizure [48]. Furthermore, acute PTZ treatment increased free fatty acid (FFA) levels, and free stearic, arachidonic, and docosahexaenoic acid levels, in the rat brain [50]. FFAs, which accumulate during epileptic seizures [53], especially arachidonic acid, can cause cell membrane and mitochondrial damage via LPO and other molecular interactions such as ATPase inhibition [50].

### 4.3. Picrotoxin-Induced Seizures

Picrotoxin (PTX) is a GABA antagonist which exerts its effect by binding to the “picrotoxin binding site”, which is closely related to the chloride ionophore in the GABA_A_ receptor complex [54]. After the administration of PTX, animals begin to develop generalized seizures characterized by clonic convulsions of the whole body [55]. PTX-induced seizures resulted in a significant increase in LPO levels in different brain structures, including the frontal cortex [47,55], striatum [47], and hippocampus in rats and mice [55]. Moreover, a reduction in GSH levels in the prefrontal cortex and the hippocampus has been observed in PTX-treated animals [47]. PTX-induced alterations in the activity of antioxidant enzymes (SOD, GPx) in the brain were reported to be region-specific [55]. The transfer of electrons between complexes I and III (complex I–III activity) was reduced in the hippocampus after PTX-induced seizures [56].

### 4.4. Classical Kindling Models

Kindled seizures generated in rats by repetitive electrical stimulation of the amygdala or hippocampus possess behavioral features similar to complex partial seizures [57]. In addition, persistent seizure susceptibility and brain alterations found in this model are comparable to those seen in human temporal lobe epilepsy (TLE) [57]. Another aspect of the amygdala-kindled rat that supports its validity is its ability to predict the protective effects of AEDs against partial seizures [27]. In the amygdala-kindled rat, LPO was increased in both hemispheres of kindled rats as compared to sham-operated controls, and cell death was also significantly increased in all hippocampal areas [58]. Increased LPO in the kindled brain correlated with hippocampal neuronal loss. Kindling reduced neuronal density in CA1, CA2/CA3 pyramidal layers, and the hilus of the dentate gyrus [58]. It has been suggested that free radicals are produced during seizures in this model, and they are involved in seizure-induced neuronal loss, however, LPO and cell loss in the hippocampus do not contribute to the development of kindled seizures [58]. Lu et al. [59] measured histopathological changes and the levels of ROS, LPO, reduced GSH, and measured activity of cytosolic cytochrome c (CytC) and caspase-3 in the hippocampus of kindled animals. They found extensive neuronal damage in the CA3 region in the kindling group, which was preceded by increases in ROS and LPO levels and a decrease in GSH, and was followed by caspase-3 activation and an increase in cytosolic CytC [59]. This study demonstrated that kindling-induced oxidative damage not only causes cell membrane destruction, but also disrupts mitochondrial function because mitochondria are involved in apoptosis by releasing apoptotic substances such as CytC from the mitochondrial intermembrane space to the cytoplasm to activate the caspase cascade [1]. The possible role played by SOD in amygdala-kindled seizures has been investigated. Increased activity of SOD in kindled brains and suppression of kindled seizures following intra-amygdaloid injection of SOD was observed in rats, suggesting that SOD participates in seizure susceptibility induced by amygdala kindling and the initiation of kindled seizures [60]. Furthermore, amygdala-kindled seizures activate the Nrf2-ARE signal pathway in the rat hippocampus [61]. The transcription factor called Nrf2 (nuclear erythroid-2-related factor 2) is referred to as the “master regulator”. It promotes the expression of numerous antioxidant, anti-inflammatory, and neuroprotective proteins [62,63]. Nrf2 binds to the antioxidant response element (ARE) to induce antioxidant and phase II detoxification enzymes under conditions of oxidative stress, which reduces oxidative stress and the accumulation of toxic metabolites [61].

### 4.5. Corneal Kindling Model

Corneal kindling is an alternative method to the electrically induced amygdala or hippocampal kindling, which requires complex surgical and EEG procedures that are not always readily available in many laboratories [64]. The corneal-kindled mouse produces a population of chronically kindled mice that can be used to screen compounds for anticonvulsant effectiveness [65]. This model, like the amygdala-kindled rat, reflects partial seizures in humans [65]. Corneal kindling in mice showed a significant increase in the levels of MDA (a product of LPO) and nitrite, and a decrease in the level of GSH in the brain tissue [66]. On the other hand, no significant changes in brain GSH levels were observed in another study, suggesting that changes in whole-brain redox status are perhaps not as important for corneal kindling [67].

### 4.6. PTZ Kindling

PTZ-induced kindling is a model of chronic epilepsy. PTZ kindling has been explored for studying epileptogenesis, epilepsy-associated comorbidities, and drug-resistant epilepsy, and has shown the ability to mimic TLE in numerous studies [29]. PTZ kindling induces oxidative stress as indicated by alterations in oxidative biomarkers. Most of them resemble alterations observed following acute PTZ-induced convulsions. An increase in LPO and decrease in GSH and antioxidant enzymes levels (SOD, CAT) were observed in both the whole brain [68,69,70,71] and in different brain regions (cortex, hippocampus) [72,73,74] of PTZ-kindled mice and rats. However, some differences between acute PTZ and PTZ kindling were observed in terms of antioxidative defense. Acute seizures of the same intensity do not cause the same alterations. The reduced SOD activity in the frontal cortex and decreased GPx activity in the hippocampus observed in the PTZ-kindled group were not present in groups exposed to a single PTZ treatment. The observed effects in these two locations are thus clearly related to the kindling phenomena rather than seizure activity [50]. It has been also demonstrated that chronic treatment with PTZ induces an increase in nitric oxide levels, as indicated by a significant rise in nitrite concentrations in the whole brain [68,69,70,71] and in separate structures [72,73]. Excessive nitric oxide generated during convulsions affects oxidative phosphorylation by inhibiting the mitochondrial respiratory enzymes, and the resistant mitochondrial dysfunction results in apoptotic neuronal death [71]. Hence, chronic PTZ administration has been documented to impair mitochondrial enzyme complex (I, II, and IV) activities [71,73]. Additionally, a significant decrease in total thiol (TSH) concentration can be observed in the brain of PTZ-kindled animals [74]. The PTZ kindling process is accompanied by numerous behavioral disturbances and related biochemical changes, including altered biomarkers of oxidative stress. In one study, the PTZ-kindled animals have shown impaired motor activity, locomotion, discrimination ability, learning, and memory, along with increased emotional tension, anxiety, hippocampal pro-inflammatory mediators (increased IL-1β and TNF-α), oxidative stress (increased TBARS, decreased GSH and CAT), and mitochondrial dysfunction [75].

PTZ kindling, as with classical kindling models, has resulted in a considerable loss of neuronal cells in the presence of oxidative/nitrative stress. This was associated with enhanced expression of inducible nitric oxide synthase (iNOS), hemeoxygenase-1 (HO-1), and vascular endothelial growth factor (VEGF), along with increased activity of thioredoxin reductase (TrxR) in hippocampal tissue [76]. Concerning neuronal damage, serum neuron-specific enolase (s-NSE), a sensitive marker of neuronal damage in several central nervous system (CNS) diseases including epilepsy, has been shown to increase in PTZ-kindled rats [10,75]. However, it was roughly proportionate to the degree of hippocampal histological damage. Therefore, it was postulated that the increased sNSE is a consequence of death in distinct neuronal populations and different brain structures [10]. In the same report, the authors carried out nuclear DNA fragmentation studies and expression studies of proapoptotic caspase 3 in the hippocampus. It has been suggested that seizures cause an early production of oxidative damage to DNA bases before significant DNA strand breaks appear, indicating that reactive oxygen species may be a contributory factor to the mechanism by which seizures cause cell death in this model [10]. According to one theory, oxidative stress can spread by causing early oxidative damage, which in turn creates additional free radicals, damages antioxidant enzymes, depletes antioxidant molecules such as GSH, and damages mitochondria, producing more ROS [77]. PTZ-induced seizures increase LPO, decrease GSH in the hippocampus, and enhance ROS generation in the mitochondria isolated from the epileptic hippocampus, indicating an aggravation of oxidative stress in epileptic kindling [77].

### 4.7. Pilocarpine Model

Pilocarpine (PILO) is a cholinergic agonist able to induce seizures and status epilepticus (SE) in rodents [78]. The PILO model reveals behavioral and electroencephalographic alterations that are similar to those in human TLE [79]. The ability of PILO to induce epilepsy depends on the activation of the m1 muscarinic receptor (m1R) [80], and some evidence suggests that, following m1R activation, seizures are maintained by activation of N-methyl D-aspartate receptors (NMDARs) [81]. The PILO model in rats is characterized by an acute phase, characterized by animals presenting long-lasting SE (12–18 h), by a seizure-free period (silent; 4–44 days, mean of 15 days), and by a spontaneous recurrent seizures period, characterizing the chronic phase [82]. It is thought that excitotoxic stimulation by PILO resulting in pathological increases in neuronal lesions in response to excess ROS production could be responsible for PILO-induced seizures [11]. Increased LPO is present in different brain structures (e.g., hippocampus [83,84,85], striatum [83,86], and frontal cortex [83,85,86]) during PILO-induced seizures, suggesting neuronal damage to these structures. However, hippocampal neuronal death and subsequent synaptic reorganization are considered to be the key changes following PILO-induced SE [79]. LPO in the hippocampus during the acute period of SE can be extended for several hours after the spontaneous recovery from SE in the PILO model [87]. Besides increased LPO, decreased reduced GSH has been implicated in neuronal death in the hippocampus after the administration of PILO [84,88]. Reduced GSH levels have also been detected in other brain regions of PILO-treated animals [86]. Nitric oxide generation and its metabolites (nitrite and nitrate), as well as their destruction by antioxidant systems, are in a steady-state balance under normal conditions [84]. A significant increase in nitrite concentration in the hippocampus has been observed after the induction of SE, suggesting an increase in concentrations of ROS involved in neuronal damage [84]. An increase in nitrite formation after PILO-induced SE has been also detected in other brain structures [86]. Concerning antioxidant enzyme levels, both increase and decrease, as well as no change in their activities, have been reported in different brain regions. Increased CAT activity and no changes in SOD activity have been found in the hippocampus of PILO-treated rats [84]. The authors of that study suggested that the hippocampus does not use SOD as the major free radical scavenging system, and probably uses CAT and GSH in this respect [84]. The involvement of CAT activity in the activation of cholinergic neurons in the hippocampus during the establishment of PILO-induced SE has been proposed in numerous studies (e.g., [89]). On the other hand, Bellissimo et al. [82] investigated SOD and GPx activity, as well as the concentration of hydroperoxides (HPxs), compounds produced as the result of phospholipid peroxidation, in the hippocampus of rats during SE, silent, and chronic periods in the PILO model. They concluded that the decreased activity of SOD associated with high HPxs concentration in animals presenting seizures indicates that this enzyme plays a major scavenger role in the hippocampus of rats submitted to the PILO model of epilepsy [82]. Some studies indicate a role for the Nrf2-ARE signaling pathway in PILO-induced seizures. Elevated mRNA expression of Nrf2 has been noted in hippocampal tissues in mice following PILO-induced SE. Three Nrf2-regulated genes, HO-1, NQO1, and mGST, showed similar expression patterns. Mice subjected to PILO treatment and injected with Nrf2 adeno-associated virus (AAV) vector had much fewer generalized seizures, as well as a significant reduction in microglia activation [63]. The oxidant-induced oxidative damage after PILO treatment was also associated with loss of vitamin E in mitochondria, as vitamin E is essential for maintaining functional integrity of mitochondria, and its level was reduced in the cerebral cortex in response to PILO-induced oxidative stress [78].

### 4.8. Kainic Acid Model

The kainic acid (kainate, KA) model is a useful animal model to investigate the development and neuropathology of TLE [87]. When the KA subtype of ionotropic glutamate receptors is activated, it causes prolonged epileptic activity in the hippocampus, followed by a specific pattern of neuropathology comparable to human TLE [11]. KA increases ROS generation and mitochondrial dysfunction, as well as apoptosis in neurons throughout the brain, notably in the hippocampal CA1 and CA3 areas and the dentate hilus [11]. Increased LPO is detected both in the whole brain [90,91] and in different brain structures (i.e., hippocampus [92,93], cerebellum [93], cortex [94,95,96], and amygdala [93]) in rodents exposed to KA-induced seizures, indicating neuronal damage in these areas. It is thought that KA-produced hydroxyl radicals cause damage to mitochondrial DNA and the increase in LPO in the brain, leading to severe seizures [91]. The generation of free radicals by KA and its correlation with neurotoxicity is a widely accepted theory [93]. Similarly to the PILO model, in the KA-induced seizures, LPO in the hippocampus during SE can be extended for several hours after the spontaneous recovery from SE [87]. LPO induced by KA could be accompanied by chronic changes in the lipid composition that could be related to the development of seizures [97]. Decreased GSH levels have also been detected in different brain regions of animals exposed to KA administration, namely, the hippocampus [92,93,98] cerebellum [93], cortex [96], and amygdala [93,98].

Concerning antioxidant enzymes, hippocampal SOD activity was shown to decrease significantly after KA administration in rats [93]. In another study, a significant reduction in GR activity in the forebrains of KA-treated rats accompanied a decrease in GSH levels [98]. Intracellular levels of GSH are maintained by GR. It has been suggested that KA-triggered ROS accumulation could inactivate GR and thus disrupt the GSH redox cycle at the enzyme level [98]. Recently, Munguía-Martínez et al. [99] studied the immunohistochemical expression and localization of the antioxidant enzymes GPx, SOD, and CAT in the brains of rats treated with KA. The results demonstrated a reduction in GPx in immunoreactive cells, although SOD and CAT immunoexpression remained unchanged for a longer time, suggesting that SOD and CAT play a more critical function and may be regarded as the principal antioxidant enzymes in the KA model [99]. Candelario-Jalil et al. [93] measured LPO, GSH, and SOD levels in different rat brain areas (i.e., hippocampus, cerebral cortex, striatum, hypothalamus, amygdala/piriform cortex, and cerebellum) after KA treatment and suggested that the pattern of oxidative injury induced by systemically administered KA seemed to be highly region-specific. It has been shown that a lower antioxidant status (GSH and SOD) does not appear to play an essential role in the selective susceptibility of particular brain areas since it correlates poorly with increases in oxidative damage markers [93]. However, other data suggest that KA induces similar oxidative stress in all of the examined brain regions (hippocampus, cortex, cerebellum and basal ganglia), and that GSH plays a major antioxidant role in the cerebral cortex but not the hippocampus [100].

Some studies have also shown a close relationship between nitric oxide and KA-induced seizures. It has been demonstrated that KA enhanced hippocampal nitric oxide generation in a severity-related manner of induced seizures in rats [101]. Both dose- and time-dependent alterations of nitric oxide levels in the temporal lobe of KA-treated mice were closely related to the development of seizure activity [102]. The KA-induced enhanced nitric oxide generation appears mainly to be involved in seizure suppression [101,102].

Examples of alterations which occur during oxidative stress induced in different tests/models of seizures and epilepsy are presented in Table 1.

## 5. Oxidative Stress: Consequence and Cause of Epileptic Seizures

As described above, oxidative stress occurs as a consequence of acute seizures or prolonged epileptic seizures (SE) and may contribute to seizure-induced brain damage. As shown in Table 1 and described in earlier sections, the changes in oxidative stress parameters relate to clearly defined convulsive activity in animal models. In the cited experimental tests of acute convulsions or in SE convulsive activity was defined as generalized tonic–clonic (tonic hindlimb extension followed by clonic convulsions) or clonic seizures. Based on these reports, it may be assumed that alterations in oxidative stress are generally associated with the convulsive final stage. However, it should be taken into account that some of the reported changes in oxidative stress may occur at earlier stages of convulsive activity. Pronounced changes in oxidative status following electroshock were observed in animals subjected to convulsive and subconvulsive stimuli (mice without tonic hindlimb extension) [43]. However, compared to tonic seizures, subconvulsive stimulation does not result in the same rapid and severe changes in the brain [43]. In this study, subconvulsive stimulation provoked a rise in GPx activity one hour later, whereas tonic convulsions caused an increase in GPx activity immediately after seizure onset [43]. Oxidative damage parameters are usually elevated immediately after the onset of convulsions and may normalize after some time, but it should be noted that this is a significant generalization. Various studies show that these changes in parameters may be delayed in time and/or long-lasting, which is partly explained by the seizure or epilepsy test used, or by different procedures of a similar test. Further, some parameters may remain diminished after convulsions as a result of the neuronal loss and hypometabolism observed in structures affected by chronic epilepsy [87]. The increase in LPO indicated by TBARS (thiobarbituric acid reactive substances) levels in the hippocampus during SE was extended for several hours after the spontaneous recovery from SE (at least 12 h) in both PILO and KA models [87]. The TBARS levels in PILO-treated rats were significantly decreased late after SE, and in KA-treated animals the TBARS returned to basal levels when measured 7–9 days or 75–80 days after SE in both models [87]. In rats subjected to multiple electroconvulsive shock, the TBARS levels were increased and CAT activity was decreased in the hippocampus and striatum 60, 90, and 120 days after the last electroshock. Additionally, SOD activity was reduced 45, 60, 90, and 120 days following electroconvulsions, indicating long lasting effects of electroconvulsions on brain oxidative parameters [41]. Despite the fact that seizure activity leads to alterations in brain oxidative stress, including long lasting ones, seizures may also cause other consequences for the brain and peripheral tissues. SE, as well as repetitive severe seizures, can induce not only cerebral but also systemic hypoxia–ischemia events, significantly affecting peripheral organs [105]. It is thought that ROS, including superoxide anions, can contribute to vascular endothelial cell dysfunction in the blood–brain barrier [105]. The impact of potential cerebral hypoxia caused by seizures on the course of seizures is a matter of debate. The brain’s requirement for oxygen and substrates increases during convulsions. Cerebral vessels dilate, systemic blood pressure rises, and cerebral blood flow increases to meet the higher metabolic needs of cerebral seizures. However, if the arterial blood is desaturated, these compensatory mechanisms cannot prevent brain hypoxia. As a result, brain lactate increases during a generalized convulsion [106]. The brain of the experimental animal has an extraordinary capacity to protect itself from the potentially harmful metabolic consequences of seizures by boosting cerebral blood flow and supplying enough oxygen to prevent cerebral hypoxia and lactic acidosis [106]. Changes in the amount of lactate and pH can also be found in blood following generalized epileptic seizures, due to local muscle hypoxia during the convulsions. The lactic acidosis that follows seizures, however, is self-limiting and does not require special treatment [107].

Another topic is whether seizure-induced oxidative stress and mitochondrial dysfunction are epileptogenic, i.e., resulting in chronic redox alterations that increase seizure susceptibility and result in the development of subsequent epilepsy [21]. Many common neurological diseases/disorders such as trauma, stroke, and tumors, can lead to oxidative stress and can be accompanied by seizures. Apart from oxidative stress, there are many pro-epileptogenic pathways of these diseases that can induce epileptogenesis (see below). A possible role of oxidative stress in the development of post-traumatic epilepsy is discussed briefly below. Furthermore, research reveals that persistent mitochondrial oxidative stress and resultant dysfunction might make the brain more susceptible to epileptic seizures. The incidence of epilepsy in mitochondrial disorders brought on by mutations in mtDNA or nuclear DNA is the most notable example of mitochondrial dysfunction causing epilepsy [21]. The myoclonic epilepsy with ragged red fibers (MERRF) syndrome is one such mitochondrial disease. It is linked to point mutations in the mitochondrial tRNALys gene [22].

There is growing evidence that epileptic seizures may be both a cause of and a consequence of neuronal cell death. The finding that intense seizure activity associated with SE can damage the hippocampal tissue due to excessive glutamate receptor activation and subsequent excitotoxicity provides proof that seizures induce brain injury [21]. In turn, epileptic seizures can occur as a consequence of traumatic brain injury (TBI). However, it is uncertain by which mechanisms brain injury leads to epileptiform network activity. TBI causes both focal and diffuse damage to the central nervous system (CNS), which can result in epileptogenesis [108]. The excitotoxic consequences that accompany a TBI are mediated by an elevated extracellular glutamate concentration brought on by neuronal overproduction and death. In the next stages of the pathophysiological molecular pathways of TBI, alteration of mitochondrial integrity is followed by the release of ROS and RNS, which together induce oxidative stress [109]. ROS cause additional damage to cells and organelles by depleting cellular antioxidants, interacting with nucleotides and proteins, and initiating peroxidation of cellular membranes. Particularly, NOX-derived ROS play a crucial role in the subsequent expansion of damage following TBI [20]. First of all, inflammation is well accepted important factor in the pathogenesis of TBI. Elevated expression of key pro-inflammatory cytokines, which have been demonstrated to exacerbate seizures, are frequently found in human and animal brain tissue after TBI [20]. The enhanced proinflammatory response impairs the integrity of the blood–brain barrier [109], which, along with elevated ROS generation, appears to be a consistent feature of experimental epileptogenic insults. It has been demonstrated in numerous in vivo studies using rodent models of TBI that an increase in glutamate response following injury modifies neuronal microcircuits, which is correlated with an increase in epileptiform activity adjacent to the site of injury [108]. In this process, oxidative-stress-related changes in mitochondrial activity cause progressive dysfunction as a part of degenerative events that trigger a vicious cycle of oxidative stress and neurodegeneration, eventually leading to post-traumatic epilepsy [108].

## 6. Oxidative Stress, Neuronal Death, and Neurodegeneration in Epileptogenesis

Epileptogenesis is thought to involve three stages: (1) the initial insult or precipitating event, (2) the latent period, and (3) the chronic epilepsy phase [110,111]. Neurogenesis, gliosis, neurodegeneration, dendritic plasticity, axonal damage or sprouting, recruitment of inflammatory cells into brain tissue, damage to the blood–brain barrier, reorganization of the extracellular matrix, and reorganization of the molecular architecture of specific neuronal cells are changes that can occur in the epileptogenic brain [112]. In terms of morphological changes in patients with epilepsy, kindled animals had comparable results. Several authors have documented neuronal damage and loss of cells or synapses, as well as the sprouting of mossy fibers, following kindling of different brain areas with electrical stimulation and after continuous perforant path stimulation [113]. Neuronal death has also been observed in PTZ-kindled animals, as manifested by pyknotic nuclei, degeneration, and necrotic nuclei in the hippocampal region of the brain [114], although morphological changes in this model occur rarely compared to other ones [113]. Administration of KA results in long-lasting recurrent limbic seizures which are followed by neuronal loss, especially in the hippocampus, amygdala, piriform cortex, and some thalamic nuclei [95]. The hippocampal regions of CA1 and CA3 and the dentate hilus are particularly affected [11].

Neuronal cell death occurs as a consequence of the excessive stimulation of the glutamatergic system. Due to the increased liberation of glutamate, there is an excessive flux of intracellular Ca^2+^ leading to cell death [113]. Excessive or persistent activation of glutamate-gated ion channels may cause neuronal degeneration in pathological conditions (e.g., neurodegenerative disorders, seizures, stroke), in which oxidative stress is a causal, or at least an ancillary, factor [115]. Thus, these two phenomena, oxidative stress and excessive activation of glutamate receptors, converge and represent sequential as well as interacting processes that provide a final common pathway for cell vulnerability in the brain [115].

Neuronal cell death and oxidative stress belong to the most essential mechanisms of epileptogenesis. Other essential mechanisms include short and long-term adaptive changes in sensitivity to GABA-ergic neurotransmission via GABA_A_ receptors, impairment of the fine-tuning mediated by dopamine (DA) receptor activity, inflammation and inflammatory processes affecting the extracellular neuronal matrix integrity, the impact of hormones, or even mechanisms of circadian rhythmicity [116]. Evidence coming from in vitro and in vivo studies shows that oxidative stress is linked to the generation of epileptiform activity and seizure-induced cell death [117]. Seizure-induced glutamate receptor activation and the ensuing calcium-dependent depolarization of the mitochondrial membrane potential cause inadequate oxygen consumption, decreased ATP synthesis, excessive ROS, nitric oxide, and peroxynitrite generation, and consequent damage to cell components including lipids, proteins, and DNA. As a result, in susceptible brain areas, impaired mitochondrial respiratory chain function and the resulting lipid peroxidation linked to seizure activity may precede neuronal injury and death in vulnerable brain areas [11].

**Figure 2:**
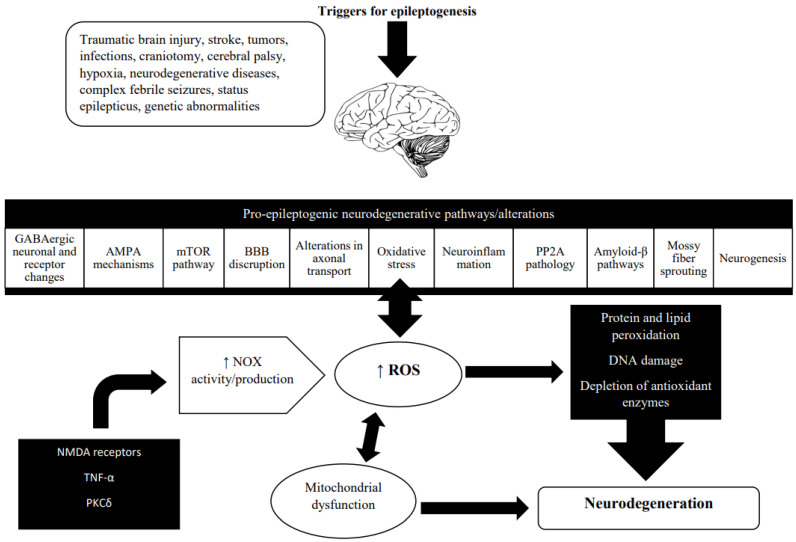
Oxidative stress and neurodegeneration in the epileptogenic brain. Neurodegeneration is a broad term that refers to the progressive changes in neuronal function that often result in neuronal death. It has been described in a wide range of brain conditions including stroke, traumatic brain injury, Alzheimer’s disease, and Parkinson’s disease. In recent years, the role of neurodegeneration in epileptogenesis has become more well-recognized. Neurodegeneration evolves during acquired epileptogenesis [118]. Neurodegenerative changes found during epileptogenesis include various molecular, cellular, and functional alterations. The main ROS producer in the injured brain microenvironment is NADPH oxidase (NOX), and growing evidence points to the involvement of NOX2 and NOX4 isoforms in epilepsy [19]. In the epileptic brain, NOX activity is triggered by the activation of NMDA receptors, an inflammatory stimulus, either on its own or via TNFα, and regulated by PKCδ and interaction with COX-2 [108]. Mitochondrial dysfunction caused by oxidative stress is known to have a significant role in neurodegeneration and epileptogenesis. Recurrent seizures cause an increase in mitochondrial ROS production, particularly superoxide, which are subsequently converted to hydroxyl species. In the presence of Cu2^+^ and Fe2^+^, the latter oxidizes lipids, proteins, and DNA, resulting in impaired neuronal metabolism and gene expression, increased membrane permeability and excitability, and, ultimately, a lower seizure threshold [7]. (Abbreviations: Gamma-aminobutyric acid, GABA; Alpha-amino-3-hydroxy-5-methyl-4-isoxazolepropionic acid, AMPA; Mechanistic target of rapamycin, mTOR; Protein phosphatase 2A, PP2A; Nicotinamide adenine dinucleotide phosphate (NADPH) oxidase, NOX; Reactive oxygen species, ROS; N-methyl-D-aspartate, NMDA; Tumor necrosis factor alpha, TNF-α; Protein kinase C delta, PKCδ; Cyclooxygenase-2, COX-2).

Neuronal death is often involved in neurodegeneration. The link between neurodegeneration and oxidative stress is presented in Figure 2.

### Seizure-Induced Oxidative Stress and Neurodegeneration: Mechanisms Involved

Acquired causes, such as a brain injury, illness, or exposure to toxic substances, can induce one or more seizures or status epilepticus (SE) [119]. Oxidative stress is rapidly triggered following SE, a neurological emergency, which is characterized by prolonged or continuous epileptic seizures, and is associated with significant morbidity and mortality [120]. In general, excessive production of ROS following SE results in LPO, DNA damage, enzyme inhibition, and mitochondrial damage, culminating in neuronal death [4]. Acute increases in ROS production and mitochondrial DNA lesion frequency are dependent on the severity of convulsive seizure activity during SE [121]. ROS and nitric oxide can react to create reactive nitrogen species. Among them is peroxynitrite, particularly toxic for cells, which is created when nitric oxide, produced by nitric oxide synthase, reacts with superoxide [122]. There is strong evidence that peroxynitrite production rises in SE, suggesting that peroxynitrite production may be a primary cause of SE-associated cell death [122]. Indeed, excessive peroxynitrite and hydrogen peroxide generation, as well as failure of protective systems such as glutathione depletion, have been considered important causes of neuronal death in many neurodegenerative diseases [123]. Furthermore, excessive ROS generation can limit mitochondrial complex I function, lower mitochondrial membrane potential, and inhibit ATP synthesis [4].

It has been assumed that mitochondria play an essential role in ROS production during seizures. It has been suggested that brain mitochondrial complex III-dependent superoxide generation can lead to seizure-related ROS formation [124]. Prolonged seizure activity, such as that seen in SE, causes mitochondrial depolarization, mitochondrial malfunction, and energy failure; therefore, although mitochondria are a key generator of ROS during short seizures, their involvement in producing ROS declines as the seizure activity continues [122]. Using robust protocols for real-time in vitro and ex vivo monitoring of ROS production during hyperexcitability, it was established that mitochondria were not the main source of ROS during SE [4]. The primary source of ROS generation during SE or prolonged seizures has been identified as NOX. Using live cell imaging techniques in glioneuronal cultures, it has been demonstrated that prolonged seizure-like activity increases ROS production primarily by NADPH oxidase and later by xanthine oxidase (XO) activity in an NMDA receptor-dependent manner. The seizure activity did not involve calcium- and mitochondria-dependent ROS production [125]. 4-(2-Aminomethyl)benzenesulfonyl fluoride hydrochloride (AEBSF), an NADPH oxidase inhibitor, suppressed significant ROS generation and subsequent neuronal cell death in the hippocampus during electrically induced SE [126]. In the pilocarpine model of epilepsy, treatment with a NOX inhibitor (apocynin) prior to induction of SE was effective in decreasing both ROS production and neurodegeneration in the rat hippocampus [127].

Despite the fact that mitochondria are not the main source of ROS during prolonged seizure activity, they are probably a key target of ROS-induced damage [122]. SE leads to excessive ROS production and consequent mitochondrial dysfunction by increasing the opening of glutamate receptors and glucose hypermetabolism. SE enhances the interactions between glutamate receptor subunits (NMDA, AMPA, and metabotropic), and increases receptor turnover and trafficking to the postsynaptic membrane, resulting in fast Ca^2+^ influx and Ca^2+^ overload. This activates many signaling pathways, causing mitochondrial swelling, a reduction in ATP, and an increase in ROS [119]. Furthermore, during SE, hypermetabolism, excessive glycolysis, and the tricarboxylic acid cycle all contribute to an increase in ROS. High lactate generation can result in cerebral lactic acidosis, which increases ROS production and causes further damage owing to mitochondrial dysfunction [119]. ROS directly affects mitochondrial function, inhibits mitochondrial complex I and III activity, decreases mitochondrial membrane potential, opens mitochondrial permeability transition pores, and inhibits ATP production [119,122]. SE-induced mitochondrial respiratory chain dysfunction results in energy failure, and can further aggravate the severity of oxidative stress and cause neuronal damage or death [120]. It is known that glutamate-mediated excitotoxicity frequently results in necrotic changes in the cerebral cortex after SE [128]. Additionally, numerous studies have revealed that apoptotic cell death plays a significant role in seizure-induced brain injury in experimental SE, and involves cysteine proteases (caspases) as well as apoptosis-inducing factor (AIF), cytochrome c, endonuclease G, Smac/DIABLO, HtrA2/OMI, and other apoptosis-related proteins [120].

After an initial period (initial insult, e.g., SE), epileptogenesis via its latent period causes the phase of chronic epilepsy [110]. During the latent phase, antioxidant mechanisms are activated and cause a decrease in the H_2_O_2_ that was generated during SE [121]. However, when neuronal excitability increases, spontaneous recurrent seizures emerge, resulting in a time-dependent increase in H_2_O_2_ due to the progressive depletion of antioxidant mechanisms (GSH, coenzyme-A-SH) [119]. This increase in ROS production causes a number of changes in brain tissue, including the accumulation of oxidized forms of antioxidant enzymes (oxidized glutathione-GSSG and coenzyme-A-SSG), mitochondrial DNA damage, protein subunit modification of excitatory ion channel proteins, inactivation of energy-dependent glutamate transporters, and a decrease in SOD and aconitase activity [119]. These changes contribute to a further increase in neuronal hyperexcitability during epileptogenesis, either directly or by increasing the amount of circulating glutamate in the synapse [119].

Experimental models of epilepsy show that the impairment of mitochondrial and tissue redox status lasts during the acute, latent, and chronic phases of epileptogenesis [121]. Electron leakage in damaged mitochondria increases the rate of superoxide formation and consequently of H_2_O_2_, and, as a result of superoxide reaction with the radical nitric oxide produced by the cellular nitric oxide synthase, peroxinitrate is generated, a stable pro-oxidant molecule with a proven role in neuronal cell damage [19]. Excessive ROS generation overwhelms the mitochondrial and extra-mitochondrial defense systems necessary for maintaining the cell’s redox homeostasis, resulting in severe oxidative stress and further mitochondrial dysfunction and ROS/RNS production. Other implications of this chain of biological processes include altered Ca^2+^ homeostasis, LPO, and membrane permeability, as well as protein and DNA damage. All of these factors eventually influence gene expression, predisposing the cell to cell cycle changes, senescence and/or cell death pathway activation [19].

## 7. Oxidative Stress and Gut Microbiota in Epilepsy

It is well established that diversified and stable microbiota enhance overall human health, whereas colonization of the gut with maladaptive and pathogenic microbiota, often known as dysbiosis, is linked to a number of disorders [129]. Among them, are neurodegenerative diseases, including Alzheimer’s disease, Parkinson’s disease, and amyotrophic lateral sclerosis, suggesting a direct or indirect communication between intestinal bacteria and the central nervous system [129]. The modes of communication include the vagus nerve, passive diffusion, and carrying by oxyhemoglobin [130]. When a microbial population is out of balance, unhealthy signals are sent to the brain, resulting in inferior situations including increased oxidative stress, unbalanced energy homeostasis, and more cellular deterioration [131]. The gut microbiota alters the central nervous system’s oxidative status by interacting with ROS levels and the antioxidant system [132]. Experimental studies reveal that modulation of the gut microbiota may have a positive impact on the epileptic brain by regulating oxidative stress and inflammation. In one study, KA-induced SE was accompanied by glial cell activation, an inflammatory response (IL-1, IL-6, and TNF- α), LPO, DNA damage, glutamate release, and a decrease in GSH level. These changes were alleviated by partial treatment with prebiotics, probiotics, and synbiotics [132]. Furthermore, probiotic supplementation exhibited protective action against PTZ-induced seizures. It alleviated PTZ-induced increases in levels of pro-inflammatory cytokines IL-1β, IL-6, and IL-17A, and restored PTZ-induced alterations in levels of oxidants, total oxidant status, and disulfide, and of antioxidants, native thiol, and total thiol in the brain tissue [133].

It should also be taken into account that many pathological conditions can accompany epilepsy and can affect its course. Some of these conditions lead to oxidative stress in various body tissues and may be accompanied by intestinal dysbiosis. An example of such a condition is obesity. Obesity is a pathologic state characterized by an excess of adipose tissue [134]. As the main metabolic regulator, normal brain function, which integrates peripheral signals, adjusts autonomic output, and controls eating behavior, is required for proper energy balance. As a result, obesity is linked to a variety of human brain illnesses [134]. It is well-documented that brain inflammation and oxidative stress are associated with obesity [135,136]. Adipose tissue undergoes immunological, metabolic, and functional alterations that cause persistent low-grade inflammation as obesity progresses [136]. It is suggested that systemic inflammation induced by obesity can drive brain inflammation, leading to a “primed” inflammatory environment in the brain [135]. On the other hand, numerous studies have shown that diet-induced obesity causes oxidative stress and mitochondrial dysfunction in the brain. Excessive dietary consumption causes the mitochondria to become overloaded with fatty acids and glucose, resulting in an increase in acetyl-CoA synthesis. This causes the Krebs cycle to produce NADH (a reduced form of nicotinamide adenine dinucleotide), which promotes an increase in the electron transfer chain in the mitochondria, and as a result, leads to ROS generation, inducing oxidative stress [136]. Furthermore, evidence is accumulating indicating that there is a link between gut microbiota and obesity risk. Specific changes in the composition and function of the human gut microbiome characterize obesity and obesity-related metabolic diseases [137]. The significance of the microbiota–gut–brain axis (MGBA) as a primary regulator of metabolism and appetite is becoming more apparent [138]. MGBA, through the gut microbiota, vagus nerve, and glucagon-like peptide-1 (GLP-1), has been proposed to play a crucial pathophysiological role in the development of obesity [138]. So far, there is no evidence that microbiota dysbiosis in people with obesity causes epilepsy. The question arises whether changes in the gut microbiome occurring in obese patients may affect their coexisting epilepsy. In general, obesity is highly prevalent in patients with epilepsy [139]. However, some studies could not identify any association between drug-resistant epilepsy and obesity [139]. On the other hand, the benefits of the ketogenic diet (KD) have been documented in the treatment of epilepsy and other neurological disorders, including Alzheimer’s disease and autism spectrum disorder, as well as in nutritional disorders (obesity) [140]. The KD is a restrictive high-fat, low protein, and very low carbohydrate diet that is typically prescribed to children with drug-resistant epilepsy [141], and is one of the diet plans that has been specifically designed to help obese people lose weight [140]. Because gut microbiome dysbiosis is linked to diseases, including epilepsy and obesity, where the KD may have therapeutic effectiveness, it has been hypothesized that KD might provide benefits by improving and restoring the gut microbiota to a premorbid condition [140]. The current evidence demonstrates that KD plays important roles in modifying the gut microbiome to ameliorate disease symptoms, mostly by increasing the *Bacteroidetes* to *Firmicutes* (B/F) ratio and decreasing *Proteobacteria* in some cases [140]. These modifications in the gut microbiome, which occur both in epileptic and obese patients, along with reported seizure reduction or weight/BMI reduction following KD, may have some therapeutic potential in obese patients with epilepsy. However, further clinical studies are required in this respect.

## 8. Antioxidant Activity of Currently Used Antiepileptic Drugs

### 8.1. Animal Studies

The majority of currently available AEDs act primarily by blocking voltage-gated ion channels (Na^+^ or Ca^2+^ channels), or activating them (K^+^ channels), increasing GABA transmission, and inhibiting glutamate excitation [141,142]. Recently, five classes of AED mechanism have been proposed: (1) modulation of voltage-gated ion channels; (2) enhancement of GABA-mediated inhibitory neurotransmission involving GABA_A_ receptors, GABA transporter 1 (GAT1), GABA-synthesizing enzyme glutamic acid decarboxylase (GAD), or GABA-metabolizing enzyme GABA transaminase; (3) inhibition of glutamate-mediated excitatory neurotransmission, mediated by ionotropic glutamate receptors; (4) modulation of neurotransmitter release via a presynaptic action including involvement of SV2A and α2δ subunit of voltage-gated Ca^2+^ channels [143,144]. The mechanism-targeted agents are represented by the fifth class, an example of which is everolimus, an inhibitor of the mechanistic target of the rapamycin (mTOR) pathway [143]. Although oxidative stress mechanisms are not thought to be the primary mechanisms responsible for available AEDs’ anticonvulsant activity, numerous experimental studies show that AEDs interact with these mechanisms. However, their anticonvulsant/antiepileptogenic potential requires further investigation, particularly in clinical conditions.

A large number of experimental studies have evaluated the effects of valproate (VPA) on oxidative stress in various animal models of seizures and epilepsy. VPA significantly attenuated the increase in MDA levels caused by electroshock [40]. Complete protection against PTZ-induced seizures was observed with VPA in rats. In PTZ-induced seizures, a significant decrease was observed in the reduced GSH levels as well as an increase in LPO in the brain. VPA treatment reversed these changes [37]. A similar mode of antioxidant activity was observed for phenytoin (PHT), phenobarbital (PB), and carbamazepine (CBZ) in MES-induced seizures in rats [145]. VPA was able to decrease the oxidative stress induced by 4-AP (4-aminopyridine), a K^+^ channel blocker, whose activity enhances the release of multiple neurotransmitters [56]. Pretreatment with VPA reduced the whole brain MDA level and increased the level of GSH and CAT in PTZ-kindled rats [10]. In PTZ-kindled mice, VPA treatment reversed oxidative damage (attenuated MDA and nitrite concentration, restored SOD, GSH, and CAT levels), and restored mitochondrial enzyme complex activity (I, II, and IV) both in the cortex and hippocampus [146].

Apart from classical AEDs, the effects of some second-generation AEDs on markers of oxidative stress during electroconvulsions have also been studied. For example, lamotrigine (LTG) significantly elevated the electroconvulsive seizure threshold, inhibited LPO, and increased brain GSH levels in acute and chronic dosages in mice [147]. In PTX-induced seizures, diazepam (DZM) and PB significantly reduced a seizure-induced increase in LPO and nitric oxide levels in the frontal cortex, and additionally, nitric oxide elevations in the hippocampus and midbrain [55].

Numerous studies on AEDs and oxidative stress have been performed with the use of the PTZ test. For example, the neuroprotective effects of DZM and gabapentin (GBP) were examined in mice against PTZ administration, which caused severe convulsions, oxidative damage (raised LPO and nitrite concentration, as well as depleted GSH, SOD, and CAT levels), and depletion of mitochondrial enzyme complex (I, II, IV) activity. Pretreatment with DZM or GBP attenuated PTZ-induced seizures, along with biochemical and mitochondrial alterations [148]. Topiramate (TPM) caused protective effects on PTZ-induced brain injury in rats by inhibiting free radical production, regulating calcium-dependent processes, and supporting the antioxidant redox system [149]. Based on experiments performed with PB, LTG, and phenazepam in PTZ-induced seizures, it has been suggested that the suppression of seizure-induced nitric oxide generation and LPO enhancement may be involved in the mechanism of action of AEDs [48]. Pregabalin (PGB) administration to PTZ-treated mice caused a significant rise in levels of GSH and GPx, and reduced NOx (total nitrate/nitrite as an indicator of nitric oxide release) and 3-NT, which was further explained on a molecular transcription basis by restoring the balance between NF-κB and Nrf2 signaling pathways [46].

The effects of several AEDs, i.e., DZM, PB, PHT, levetiracetam (LEV), PGB, TPM, and felbamate (FBM), on the course of PTZ-induced kindling and oxidative stress markers in mice have been investigated. Treatment with LEV, PB, and PGB significantly reduced the brain nitric oxide levels in PTZ-kindled animals. Both the brain peroxide and TBARS levels were found to be decreased in DZM, LEV, FBM, and TPM-treated groups compared to the PTZ kindling control group. Among the animals that received AEDs treatments, LEV-, PB-, and TPM-treated groups showed significantly elevated brain GSH levels, and LEV-, TPM-, and FBM-treated mice exhibited an increase in SOD activity. Only LEV and FBM significantly terminated the protein peroxidation when compared with the PTZ control group, as indicated by a decrease in protein carbonyl levels [150]. In another study on the PTZ kindling model in mice, LEV reduced nitrite levels in the hippocampus and LPO in the striatum and prefrontal cortex, while it increased reduced GSH in the cortex [151]. The effects of lacosamide (LCM) have also been investigated on biochemical and mitochondrial parameters after PTZ kindling in mice. The hippocampi were collected to evaluate free radicals, the activities of SOD, CAT, and the mitochondrial complexes I–III, II, and II–III. Additionally, Bcl-2 and cyclo-oxygenase-2 (COX-2) expressions were measured in the hippocampus. LCM decreased free radicals and SOD activity and was able to increase CAT activity. LCM improved the enzymatic mitochondrial activity of complex I-III and the activity of complex II [152].

It is well documented that LPO, nitrite/nitrate formation, and changes in antioxidant brain enzymes are involved in the pathophysiology of PILO-induced seizures and SE [153]. Experimental data show that LEV [153], VPA [154], and PHT [155] display activity against PILO-induced oxidative stress in the hippocampus [153]. Numerous studies have documented the protective effects of AEDs against the neurotoxicity and oxidative stress caused by KA. For instance, in rats, LEV markedly controlled KA-induced seizures, decreased LPO and prevented the brain loss of GSH in the cortex and diencephalon in rats [96]. TPM significantly reduced the KA-induced LPO in the piriform cortex and frontal cortex [95].

### 8.2. Clinical Studies

Parameters of oxidative stress (erythrocyte membrane LPO, erythrocyte enzymes SOD, GPx, GR, CAT, and plasma vitamin C, vitamin E, vitamin A, and ceruloplasmin activities) were determined in patients with epilepsy having generalized seizures. Compared to the control group, LPO was higher, whereas GR and plasma vitamin C and A concentrations were significantly lower. The low antioxidant status in the blood of the patients improved after treatment with PB, suggesting that free radicals may be implicated in epilepsy [156]. The effect of AEDs on plasma concentrations of total glutathione (tGSH), including reduced (GSH) and oxidized (GSSG) forms, in patients with epilepsy seems to be drug-dependent. Plasma tGSH concentrations were significantly lower in patients treated with CBZ or PHT and did not differ significantly from those in controls when measured in patients treated with VPA or PB [157]. During the treatment with VPA or CBZ of children suffering from different types of seizure/epilepsy (e.g., tonic–clonic seizures, juvenile myoclonic seizures, absence seizures, childhood epilepsy with occipital paroxysm, and TLE), the erythrocyte GPx and GSH levels were increased and decreased, respectively, as compared to those of healthy control subjects [158]. In another study, an increase in MDA and GPx levels and a decrease in total antioxidant capacity were observed in patients treated with VPA in particular [159]. The balance of parameters, including those of oxidative stress/antioxidant systems, differs between patients with epilepsy and healthy controls. They have been correlated with seizure pathophysiology and their degree of control or resistance to antiepileptic drug therapy [159].

## 9. Antioxidants with Potential Anticonvulsant/Antiepileptogenic Effects

Many compounds have been studied for their ability to reduce oxidative stress in animal models of seizures and epilepsy, examples of which are presented in Table 2.

Antioxidants (e.g., vitamin C or E, melatonin, resveratrol) have been documented to exert anticonvulsant effects in various models of experimental seizures [7]. An important question arises whether antioxidants may also exhibit antiepileptogenic properties. Considering that oxidative stress may participate in the mechanisms of epileptogenesis (see above), the protective activity of antioxidants seems probable. Indeed, resveratrol significantly prevented spontaneous seizure activity in rats surviving status epilepticus induced by intrahippocampal kainate [111]. Moreover, in resveratrol-pretreated animals, the degree of neurodegeneration in the hippocampus was evidently lower and mossy fiber sprouting was distinctly reduced when compared to the control rats. The antiepileptogenic activity of resveratrol was also confirmed in PTZ-kindled rats, because, in comparison with the control group, the resveratrol-treated animals exhibited extended seizure latency and reduced seizure score accompanied with neuroprotection in vulnerable brain structures and inhibition of oxidative stress [111]. Encouraging results were obtained with a combination of two antioxidants: sulforaphane (a bioactive phytochemical) and N-acetylcysteine. This combination significantly inhibited epileptogenesis in rats exposed to status epilepticus evoked by the electrical stimulation of the ventral hippocampus. Spontaneous seizure frequency was distinctly reduced when evaluated 5 months after status epilepticus. Additionally, a clearcut neuroprotective effect was noted in the hippocampus, and the cognitive performance of animals receiving the combined treatment was considerably better [111].

There are not many clinical data on the use of antioxidants in people with epilepsy. According to certain clinical findings, some antioxidant agents might be employed as adjuvants in the treatment of drug-resistant epilepsy. Melatonin, vitamin E, selenium, and allopurinol are a few examples [164]. In a randomized, double-blind, placebo-controlled study, in 24 children with pharmacoresistant epilepsy having generalized tonic–clonic and other types of seizures, the addition of D-α-tocopheryl acetate (vitamin E 400 IU/day) to existing AEDs was accompanied by a significant reduction in seizures in 10 of 12 cases [165]. However, in another clinical trial, no significant change in seizure frequency was observed when vitamin E and a placebo were compared as add-on therapy in 43 patients ≥ 12 years of age with uncontrolled focal or generalized seizures [166]. In a clinical pilot study, Yürekli and Naziroğlu [167] examined the effects of supplementation with a combination of TPM and selenium (Se) on antioxidant and oxidant values in patients with epilepsy and pharmacoresistant epilepsy. The erythrocyte and plasma total antioxidant status, erythrocyte GSH and GPx, and plasma vitamins A and C were increased either by Se or Se + TPM in groups with epilepsy and refractory epilepsy. Seizure numbers were decreased in groups of patients supplemented with TPM and TPM + Se [167]. Recently, a randomized, double-blind, add-on placebo-controlled clinical trial was conducted to evaluate the effect of add-on melatonin in the treatment of generalized epilepsy with generalized onset motor seizure in adults. The test group received a combination of add-on melatonin with VPA. The responder rate and seizure-free rate were higher in the test group compared to the control group, and melatonin increased serum GR level [168]. However, other trials with good methodological quality are required to evaluate the effectiveness and tolerability of melatonin as an add-on treatment for epilepsy [169].

## 10. Conclusions

Oxidative stress, resulting from excessive ROS production and insufficient antioxidant defense has been associated with cardiovascular or inflammatory diseases, cancer, and neurodegenerative diseases (e.g., Alzheimer’s disease). Accumulated evidence also seems to suggest an involvement of oxidative stress in the pathophysiology of epileptogenesis and subsequent epilepsy. The detailed mechanisms linking the initial brain insult with epileptogenesis have not been fully elucidated. However, glutamate excitotoxicity, neuroinflammation, and oxidative stress (a pathogenic triad) seem to be involved in the neurobiology of neurodegenerative brain diseases, including epilepsy.

Generally, experimental seizures result in an increase in LPO and a reduction in the activity of antioxidant enzymes. A good example is pilocarpine-induced status epilepticus, which shares these features and leads to diffuse neurodegeneration in rat brains [79]. There are convincing data linking neuronal death in this seizure model with oxidative stress [84,88]. Subsequent spontaneous seizures following pilocarpine-induced status epilepticus after a silent period [31] indicate that a process of epileptogenesis was initiated, and that oxidative stress might be one of the important factors involved.

Some AEDs possess antioxidant properties. For instance, valproate prevented electroconvulsion-induced elevation of MDA concentration [40], PTZ-induced decrease in reduced GSH level, and PTZ-induced increase in LPO levels in the brain. Similar activities were also evident for carbamazepine, phenobarbital, and phenytoin, as evaluated with MES-induced convulsions in rats.

The antiepileptogenic potential of some antioxidants needs to be particularly underlined. Considering that, e.g., resveratrol may possess a number of mechanisms of action, including a negative modulation of NMDA and kainate receptors [7], the involvement of its antioxidative properties in the inhibition of epileptogenesis may not be crucial. However, this antioxidant elevates the activity of antioxidant enzymes and reduces the generation of free radicals. Additionally, it produces an anti-inflammatory effect as free radicals are involved in promoting inflammatory responses [111]. Probably, all these effects may be involved in the antiepileptogenic activity of resveratrol. Sulforaphane has been documented to enhance the expression of Nrf2 in the nucleus in vivo, which increases the transcription of some antioxidant response genes and reduces the concentration of proinflammatory cytokines [111]. N-acetylcysteine, being a direct antioxidant, adds an additional mechanism to the combined treatment with sulforaphane, which results in the antiepileptogenic activity. In addition, the NOX2 competitive inhibitor, gp91ds-tat, has been documented to efficiently reduce ROS generation in rats following KA-induced SE as well as KA-induced epileptiform activity in vitro [170]. Furthermore, the above-mentioned NOX inhibitor apocynin exhibited anticonvulsant activity against PTZ-induced kindled seizures and reduced PTZ-evoked ROS production [171]. A possibility thus arises that antioxidative compounds may be regarded as targets for the inhibition of epileptogenesis and seizures.

Clinical studies on the use of antioxidants as adjuvants in the treatment of epilepsy have yielded ambiguous results. The numbers of patients participating in the studies were quite low, and so further, preferably randomized, trials are required to answer the question of whether an add-on therapy with antioxidants may potentiate the anticonvulsant efficacy of AEDs. Nevertheless, antioxidants, due to their neuroprotective activity in animal studies, may prevent neurodegeneration in patients with epilepsy.

## Figures and Tables

**Table 1:** Selected alterations in oxidative stress in different animal models of seizures and epilepsy. (Abbreviations: Pentylenetetrazole, PTZ; Lipid peroxidation, LPO; Superoxide dismutase, SOD; Glutathione peroxidase, GPx; Glutathione reductase, GR; Catalase, CAT; Glutathione, GSH; Glutathione disulfide, GSSG; Non-protein mixed disulfides, NPSSR; Cysteine, CSH; Non-protein/cysteine mixed disulfides, NPSSC; Protein thiols, PSH; Protein symmetric disulfides, PSSP; Protein/cysteine mixed disulfides, PSSC; Protein/non-protein mixed disulfides, PSSR; Nitric oxide, NO; Total thiols, TSH; ↑, increased; ↓, decreased).

Alterations in Oxidative Stress	Seizures	Animals	Effects	Brain Region	Ref.
**Lipid peroxidation**	**Electroconvulsions**	Mice	↑ LPO	Whole brain	[35]
		Rats	↑ LPO	Whole brain	[36,37,38]
		Rats	↑ LPO	Frontal cortex	[34]
		Rats	↑ LPO	Cortex	[39]
		Mice	↑ LPO	Cortex	[40]
	**PTZ-induced seizures**	Rats	↑ LPO	Whole brain	[36,37,38]
		Rats	↑ LPO	Cortex	[48,49,103]
		Mice	↑ LPO	Prefrontal cortex, hippocampus, striatum	[47]
	**Picrotoxin-induced seizures**	Mice	↑ LPO	Prefrontal cortex, striatum	[47]
		Rats	↑ LPO	Frontal cortex, hippocampus, midbrain	[55]
	**PTZ kindling**	Mice	↑ LPO	Whole brain	[68,69,70,71]
		Mice	↑ LPO	Cortex, hippocampus	[73]
		Rats	↑ LPO	Temporal cortex, hippocampus	[74]
		Rats	↑ LPO	Hippocampus	[75]
	**Amygdala kindling**	Rats	↑ LPO	Cortex	[58]
		Rats	↑ LPO	Hippocampus	[59]
	**Pilocarpine-induced seizures**	Rats	↑ LPO	Hippocampus	[84,87,88,104]
		Rats	↑ LPO	Hippocampus, striatum, frontal cortex	[83]
		Rats	↑ LPO	Cortex, hippocampus	[85]
		Rats	↑ LPO	Cortex	[78]
		Rats	↑ LPO	Striatum, frontal cortex	[86]
	**Kainate-induced seizures**	Rats	↑ LPO	Whole brain	[90]
		Mice	↑ LPO	Whole brain	[91]
		Rats	↑ LPO	Hippocampus	[87]
		Rats	↑ LPO	Cortex	[95]
		Rats	↑ LPO	Cortex, diencephalon	[96]
		Mice	↑ LPO	Cortex	[94]
**Lipid peroxidation**	**Electroconvulsions**	Rats	↓ LPO	Hippocampus	[42]
**Alterations in the activity of antioxidant enzymes**	**Electroconvulsions**	Rats	↓ SOD, GPx, GR, CAT	Whole brain	[36,38]
		Rats	↓ SOD, GPx	Frontal cortex, hippocampus, cerebellum, pons-medulla	[44]
		Rats	↓ SOD, CAT	Hippocampus, striatum	[41]
		Rats	↑ SOD, CAT	Hippocampus	[42]
		Rats	↑ SOD, GPx	Hippocampus, cerebellum	[34]
	**PTZ-induced seizures**	Rats	↓ SOD, GPx, GR, CAT	Whole brain	[36,38]
		Mice	↓ CAT	Hippocampus	[45]
		Rats	↓ GPx	Frontal cortex, cerebellum	[50]
	**Picrotoxin-induced seizures**	Mice	↓ CAT	Hippocampus	[56]
	**PTZ kindling**	Mice	↓ SOD	Whole brain	[68,71]
		Mice	↓ CAT	Whole brain	[70,71]
		Mice	↓ CAT	Cortex, hippocampus	[73]
		Rats	↓ SOD, CAT	Temporal cortex, hippocampus	[74]
		Rats	↓ CAT	Hippocampus	[75]
		Rats	↓ GPx	Frontal cortex, hippocampus, cerebellum, pons-medulla	[50]
		Rats	↓ SOD	Frontal cortex	[50]
	**Amygdala kindling**	Rats	↑ SOD	Whole brain	[60]
	**Pilocarpine-induced seizures**	Rats	↑ CAT	Hippocampus	[84,89,104]
		Rats	↑ CAT	Striatum, frontal cortex	[86]
		Rats	↑ SOD, CAT, GPx	Cortex	[78]
		Rats	↑ SOD, CAT	Hippocampus	[85]
	**Kainate-induced seizures**	Rats	↓ SOD	Hippocampus	[93]
		Rats	↓ GR	Forebrain	[98]
**Alterations in the thiol redox state**	**Electroconvulsions**	Rats	↓ GSH	Whole brain	[37]
	**PTZ-induced seizures**	Rats	↓ GSH	Whole brain	[37]
		Mice	↓ GSH	Hippocampus	[45]
		Mice	↓ GSH, GSSG, CSH, NPSSC, PSSR and PSSC ↑ protein carbonyl, PSSP	Cortex	[51]
		Mice	↓ PSH, CSH and NPSSC, ↑ PSSP, NPSSR	Hippocampus	[52]
	**Picrotoxin-induced seizures**	Mice	↓ GSH	Prefrontal cortex, hippocampus	[47]
	**PTZ kindling**	Mice	↓ GSH	Whole brain	[68,69,70,71]
		Mice	↓ GSH	Cortex, hippocampus	[73]
		Rats	↓ GSH, TSH	Temporal cortex, hippocampus	[74]
		Rats	↓ GSH	Hippocampus	[75]
	**Amygdala kindling**	Rats	↓ GSH	Hippocampus	[59]
	**Pilocarpine-induced seizures**	Rats	↓ GSH	Hippocampus	[83,84]
		Rats	↓ GSH	Striatum, frontal cortex	[86]
	**Kainate-induced seizures**	Rats	↓ GSH	Cortex, diencephalon	[96]
		Rats	↓ GSH	Forebrain	[98]
		Rats	↓ GSH	Hippocampus	[92]
		Rats	↓ GSH	Hippocampus, amygdala/piriform cortex, cerebellum	[93]
**NO production**	**PTZ-induced seizures**	Rats	↑ NO	Cortex	[48]
		Mice	↑ NO	Hippocampus	[46]
	**Picrotoxin-induced seizures**	Rats	↑ NO	Frontal cortex, hippocampus, midbrain	[55]
	**PTZ kindling**	Mice	↑ NO	Whole brain	[68,69,70,71]
	**Pilocarpine-induced seizures**	Rats	↑ NO	Hippocampus, striatum, frontal cortex	[83]
		Rats	↑ NO	Striatum, frontal cortex	[86]
**Vitamin E concentration**	**PTZ-induced seizures**	Rats	↓ Vitamin E	Cortex	[49,103]
	**Pilocarpine-induced seizures**	Rats	↓ Vitamin E	Cortex	[78]
**Vitamin C concentration**	**PTZ-induced seizures**	Rats	↓ Vitamin C	Cortex	[103]

**Table 2:** Effects of selected antioxidants/antioxidative agents in animal models of seizures and epilepsy. (Abbreviations: Pentylenetetrazol, PTZ; Lipid peroxidation, LPO; Superoxide dismutase, SOD; Glutathione peroxidase, GPx; Catalase, CAT; Glutathione, GSH; Nitric oxide, NO; malondialdehyde, MDA; Nuclear erythroid-2-related factor 2, Nrf2).

Antioxidant	Animal Test/Model	Animals	Anticonvulsant Effect	Antioxidant Effect in the Brain	Ref.
α-tocopherol (vitamin E)	PTZ-induced seizures	Rats	Decreased the intensity of clonic seizures	Lowered the PTZ-induced increase in LPO; reduced NO generation	[48]
	Pilocarpine model	Rats	Decreased the percentage of convulsive animals, increased latency to the first seizure, improved the survival rate	Produced an increase in CAT activity	[89]
Ascorbic acid (Vitamin C)	Pilocarpine model	Rats	Increase the latency to the first seizure and decreased the mortality rate	Decreased LPO levels and increased CAT activity in the hippocampus	[103]
	Pilocarpine model	Rats	Reduced the percentage of convulsive animals, increased latency to the first seizure and improved the survival percentage	Decreased LPO levels and nitrite content in the hippocampus	[88]
Sulforaphane (Nrf2 activator)	6 Hz, fluorothyl, and pilocarpine model	Mice	Elevated the seizure thresholds to 6 Hz stimulation and fluorothyl, and protected mice against pilocarpine-induced seizures	Increased CAT and SOD activity, and abilities of hippocampal mitochondria to produce ATP	[160]
Dimethyl fumarate (Nrf2 activator)	PTZ kindling model	Rats	Decreased the number of kindled animals	Improved the levels of GPx, SOD, and GSH; reduced LPO	[161]
Melatonin	Kainate model	Mice	Abolished seizures	Attenuated LPO, prevented damage to mitochondrial DNA in the frontal and middle cortex	[91]
Hesperidin	PTZ-induced seizures	Mice	Attenuated PTZ-induced seizures and potentiated the anticonvulsant activity of diazepam and gabapentin	Attenuated LPO and nitrite concentration, showed protection against depletion in brain SOD, GSH and CAT levels, restored mitochondrial enzyme complex (I, II, and IV) activity	[148]
Hesperidin	PTZ kindling	Mice	Reduced the course of kindling	Attenuated alterations in LPO, nitrate, GSH and antioxidant enzyme levels (SOD, CAT) and mitochondrial complex (I, II, and IV) activities	[71]
Resveratrol	PTZ kindling	Rats	Increased the latency to myoclonic jerks, clonic seizures, and generalized tonic–clonic seizures, improved the seizure score and decreased the number of myoclonic jerks	Reduced the whole brain MDA level and increased the levels of GSH and CAT, decreased the expression of caspase 3 in the hippocampus	[10]
Resveratrol	Kainate model	Rats	Reduced the incidence of convulsions	Attenuated LPO levels	[90]
Coenzyme Q10	PTZ kindling	Mice	Attenuated kindling score	Attenuated LPO, and nitrite concentration and restored GSH, SOD and CAT levels in the hippocampus and cortex, restored mitochondrial enzyme complex (I, II and IV) activities	[73]
Curcumin	PTZ kindling	Rats	Increased the latency to myoclonic jerks, clonic seizures as well as generalized tonic–clonic seizures, improved the seizure score and decreased the number of myoclonic jerks	Attenuated the increased MDA levels, reversed the decreased brain GSH levels	[162]
Lipoic acid	Pilocarpine model	Rats	Reduced the percentage of convulsive animals, increased latency to the first seizure, increased the survival rate	Reduced LPO level and nitrite content as well as increased SOD and CAT activities in the hippocampus	[163]
